# Trends in Prevalence of HIV-1 Drug Resistance in a Public Clinic in Maputo, Mozambique

**DOI:** 10.1371/journal.pone.0130580

**Published:** 2015-07-07

**Authors:** Dulce Celina Adolfo Bila, Lídia Teodoro Boullosa, Adolfo Salvador Vubil, Nédio Jonas Mabunda, Celina Monteiro Abreu, Nalia Ismael, Ilesh Vinodrai Jani, Amilcar Tanuri

**Affiliations:** 1 National Institute of Health of Mozambique, Maputo, Mozambique; 2 Department of Genetic, Molecular Virology Laboratory, Federal University of Rio de Janeiro, Rio de Janeiro, Brazil; 3 Department of Institute of Biophysics Carlos Chagas Filho, Federal University of Rio de Janeiro, Rio de Janeiro, Brazil; University of Pittsburgh, UNITED STATES

## Abstract

**Background:**

An observational study was conducted in Maputo, Mozambique, to investigate trends in prevalence of HIV drug resistance (HIVDR) in antiretroviral (ART) naïve subjects initiating highly active antiretroviral treatment (HAART).

**Methodology/Principal Findings:**

To evaluate the pattern of drug resistance mutations (DRMs) found in adults on ART failing first-line HAART [patients with detectable viral load (VL)]. Untreated subjects [Group 1 (G1; n=99)] and 274 treated subjects with variable length of exposure to ARV´s [6–12 months, Group 2 (G2;n=93); 12-24 months, Group 3 (G3;n=81); >24 months (G4;n=100)] were enrolled. Virological and immunological failure (VF and IF) were measured based on viral load (VL) and T lymphocyte CD4+ cells (TCD4+) count and genotypic resistance was also performed. Major subtype found was C (untreated: n=66, 97,06%; treated: n=36, 91.7%). Maximum virological suppression was observed in G3, and significant differences intragroup were observed between VF and IF in G4 (p=0.022). Intergroup differences were observed between G3 and G4 for VF (p=0.023) and IF between G2 and G4 (p=0.0018). Viral suppression (<50 copies/ml) ranged from 84.9% to 90.1%, and concordant VL and DRM ranged from 25% to 57%. WHO cut-off for determining VF as given by 2010 guidelines (>5000 copies/ml) identified 50% of subjects carrying DRM compared to 100% when lower VL cut-off was used (<50 copies/ml). Length of exposure to ARVs was directly proportional to the complexity of DRM patterns. In Mozambique, VL suppression was achieved in 76% of individuals after 24 months on HAART. This is in agreement with WHO target for HIVDR prevention target (70%).

**Conclusions:**

We demonstrated that the best way to determine therapeutic failure is VL compared to CD4 counts. The rationalized use of VL testing is needed to ensure timely detection of treatment failures preventing the occurrence of TDR and new infections.

## Introduction

Mozambique, a sub-Saharan country with HIV prevalence of 11.5% [1], provides highly active antiretroviral therapy (HAART) based on a public health approach [2,3]. The antiretroviral (ARV) program was introduced in 2003 and was initially mainly provided at the capital city, Maputo. Rapid scale-up accompanied by decentralization and integration of HIV care within primary care services, resulted in 308.578 people being put on HAART by December 2012 [4].

Treatment options are based on WHO guidelines for treating HIV infected people in low income countries. First-line regimen comprises two NRTI [stavudine/zidovudine and lamivudine (d4T/AZT and 3TC)] and one NNRTI [nevirapine/efavirenz (NVP/EFV)] whereas a PI based HAART is used for second line regimen mostly composed by Aluvia (LPV boosted RTV). Monitoring of treatment is performed using immunological parameters (CD4+ T lymphocyte counts) and clinical criteria [2,3,5].

The effectiveness of first-line therapies in decreasing morbidity and mortality has been documented in several reports [6–9]. However, such reductions can be undermined by virological failure caused by appearance of resistance associated mutations (RAM´s) due to the lack of adherence as well pharmacogenetic individual variations. RAMs are produced by lack of proofreading exonuclease activity of the HIV reverse transcriptase (RT). Selection of resistant variants despite the use of effective HAART regimens, aggravated by the low genetic barrier of some drugs, can lead to the establishment of drug resistance viral population in treated individuals [10–12].

Effective ARV programs rely on the maintenance of sustainable viral suppression preventing the occurrence of both new infections and transmission of drug resistance (TDR) strains as well vertical transmission of HIV+ pregnant women [13]. Although some short-term studies suggest little difference in therapy response in patients carrying non-B subtypes from that of patients infected with subtype B, other studies showed a significant difference in responses to treatment for different subtypes. Limited and conflicting evidence comes from work done on non-B subtypes where different studies have shown characteristic subtype C polymorphic sites in HIV-1 in RT region can lead to different mutation profiles such as V106M selected by efavirenz in subtype C and it is very rare in B counterpart [14]. Various studies done in countries where non-B subtypes dominated epidemics have also revealed differences in frequencies of TAM mutations observed in subjects failing first-line therapy. Discrepancies were also seen in frequencies of development of K65R mutation after failing Frist-line regimens composed by d4T and AZT [15–18]. This important mutation is also more frequent in subtype C individuals failing tenofovir as First-line therapy [19,20]. This fact can really impact the usage of tenofovir in PreP interventions.

Cross resistance to other NNRTI´s, including next generation inhibitors, is seen with mutation Y181C resulting from a change from tyrosine to cysteine at position 181 [15]. In a context where viral load is not offered routinely to monitor treatment and this is done solely based on immunological and clinical criteria. Knowledge of trends in prevalence of HIVDR in ART-naïve adults initiating ART and virological outcomes from individuals receiving first-line therapy is of great importance to better monitor the effectiveness of the ARV treatment in a long run. This information will direct decision makers on the choice of first-line and second-line options in the country thus preventing individuals from remaining on failing therapies that will result in the development of more complex patterns of RAMs and increase the transmission of resistant strains of the virus. Data generated by studies on primary and acquired HIVDR will inform on the efficacy of national algorithms for detection and management of suspected treatment failure and efficient strategies for introduction of rationalized VL testing. The aim of this study is to define the proportion of pre-treatment individuas carry RAMs. Additionaly, we will calculate the proportion of adults on ART for 6–12, 12–24 and >24 months failing 1^st^ line using, viral load measurement comparing to CD4 response, and defining RAMs profile.

## Materials and Methods

### Study population

The study was conducted at Alto Maé Health Centre at the capital Maputo, one of the first sites to provide HAART in the country. Study group consisted of drug naive HIV positive adult subjects and subjects on first-line treatment for different periods (> 6 months). Pregnancy, low adherence and active opportunistic infections were the main exclusion criteria. Virological failure was defined as viral load >50 copies/ml immunological failures was primarily defined as TCD4+ count below 200 cells/mm^3^ following the country ARV guideline. In order to compare the virological significance of viral load cutoff above and below 5000 copies/ml, two cut-off points for virological failure (VF) were defined in this study: viral load ≤50 copies/ml (study cutoff) and ≤5000 copies/ml (WHO cutoff, given by 2009 guidelines).

### Ethics approval

Informed written consent was given to all potential participants, and signed by consenting subjects. Study protocol, including consent procedures were approved by the appropriate national ethics review committee in Mozambique.

### Specimen collection and processing

After getting written informed consent, ten milliliters of whole blood were drawn from each patient in EDTA tubes and sent to central laboratory at National Institute of Health, Maputo, Mozambique, for analysis. TCD4+ counting was performed using150ul of blood using Becton Dickinson (BD) FACSCalibur (Becton-Dickison, Fraklin Lakes, NJ), and these were performed at HIV Reference Laboratory in Mozambique. Buffy coat and plasma were separated from remnant blood and stored at -80°C and latter shipped to Laboratório de Virologia Molecular, Universidade Federal do Rio de Janeiro, Brazil, were genotyping was performed. HIV-1 viral load was quantified using COBAS TaqMan48 (Roche Diagnostics, USA), at the HIV Reference laboratory in Mozambique. Genomic DNA was extracted using QIAamp blood DNA extraction kit (QIAGEN, Germany), following manufacturers manual. Nested PCR was used to amplify a 1000bp fragment of the *pol* gene spanning the complete PR (297bp) and the polymerase domain of the RT(703bp). Both PCR and sequencing conditions have been described elsewhere [20,21]

### Sequence Analysis

*Pol sequences* were aligned using BioEdit sequence alignment editor version 7.0.5.2. Genotypic resistance interpretation was performed using HIVdb program from Stanford database Version 6.1.1 [22]. HIV-1 subtypes were inferred using REGA HIV-1 Subtyping Tool, Version 2.0 and a neighbor joining philogenetic tree constructed using kimura 2-parameter model using MEGA Version 4.0 [23]. Sequence recombination was predicted using jpHMM tool [24]. Drug susceptibility for HIV-1 sequences with ≥ 1RAM was predicted using Stanford algorithm, version 6.2.0.

### Statistical Analysis

Intergroup differences in viral load and TCD4+ count were determined using usando Wilcoxon rank sum test with p-value adjusted with Bonferroni test. Intragroup statistical significance of differences between IF and VF were also assessed using *Mann-Whitney* test. Differences were considered statistically significant at *P* < 0.5 without correction. All statistical analysis was performed using the statistical program R.

## Results

### Population, virological and immunological description

The study enrolled 373 HIV+ subjects from December 2009 to August 2010 assisted a health center located in Maputo, Mozambique. Study population was divided into two groups of untreated [Group 1 (G1; n = 99)] and treated subjects. Treated subjects group was further subdivided into three different subgroups according to length of exposure to ARV´s [6–12 months, Group 2 (G2; n = 93); 12–24 months, Group 3 (G3; n = 81); 24< months (G4; n = 100)]. Viral suppression bellow WHO cutoff was 92.5%, 96.3% and 87% for G2, G3 and G4, respectively. When undetectability of virus load was utilized as cutoff, a viral suppression of 85%, 90.1% and 76% was observed for the same groups, respectively. The theraputical failure measured by CD4 response did not agreed with individuals failing by virological parameters. In fact, there was a noticeable number of patients not increasing the CD4 counts after HAART initiation (Immunological non-responders, INR) showing undetectable VL in all treated groups analyzed (Table 1). Of note, the number of INR decreased in G4 with the increment of VF.

**Table 1:** Epidemiological and clinical data from patients participating in the study.

Characteristic	Naive	G2	G3	G4
	n = 99	n = 93	n = 81	n = 100
Gender				
Male n(%)	28 (28.3)	33 (34.4)	25 (30.9)	34 (34)
Female n(%)	71 (71.7)	61 (65.6)	56 (69.1)	66 (66)
CD4^+^ cell count (cells/μl)				
<200 n(%)	13 (13.1)	19 (20.4)	18 (22.2)	11 (11)
≥201 n(%)	86 (86.9)	74 (79.6)	61 (77.8)	89 (89)
HIV RNA (copies/ml)				
<50 n(%)	5 (5.05)	79 (85.0)	73 (90.1)	76 (76.0)
51–5000 n(%)	35 (35.35)	**7 (7.5)**	**5 (6.2)**	**11 (11.0)**
		**0 (0)**	**(100)**	**4 (40)**
≥5000 n(%)	59 (59.60)	**7 (7.5)**	**3 (3.7)**	**13 (13.0)**
		**2 (100)**	**(0)**	**6 (60)**
INR* n(%)	NA	12 (12.1)	15 (18.5)	5(5.1)

* Immunological non-responders

### Primary resistance

In total, 68 sequences from naïve subjects with detectable viral load were sequenced (72.3%) and these mainly clustered with subtype C (97.06%; n = 66), with the occurrence of one B/D (1.47%) and one C/G (1.47%) mosaics. Similarly, a subtype C dominance was observed in sequences from subjects failing treatment with the occurrence of one sequence with subtype A (1.5%), one with subtype B (1.5%), as well a mosaic B/C (1.5%). We could also found one divergent sequence (1.5%) that could not be resolved by phylogenetic analysis(Data not shown). The overall prevalence of primary drug resistance based on WHO mutation list was 0%, 4.4% and 1.5% for NRTI, NNRTI and PI respectively.

**Fig 1:**
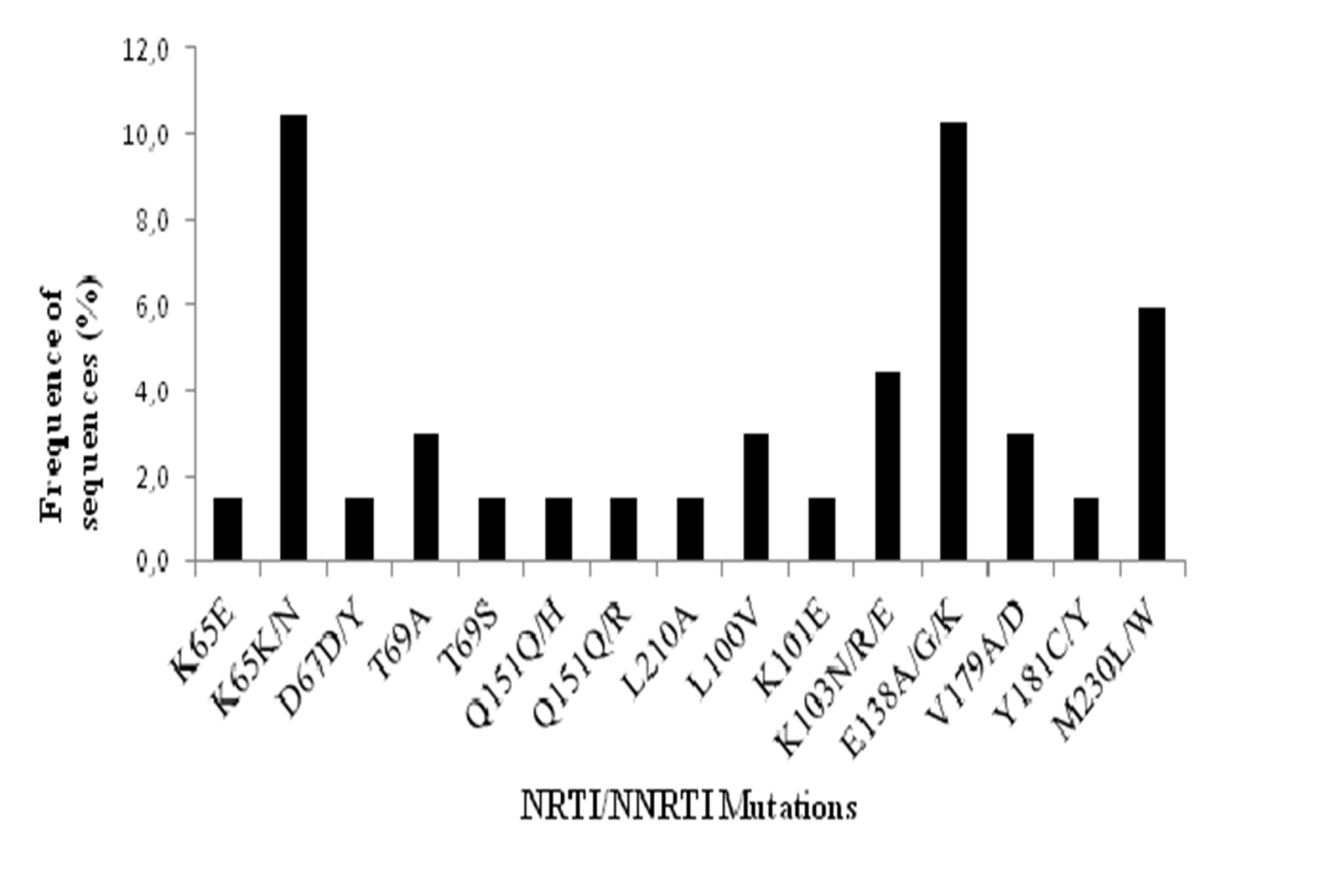
Prevalence of HIV-1 RAM and aminoacid substitutions at codon positions known to be associated to resistance to NRTI´s (a) and NNRTI´s (b) in 68 sequences from naïve subjects, according to IAS-USA 2011 list.

As previously reported for subtype C, the prevalence of second generation NNRTI mutation E138A/G/K (8.8%) and M230L (5.9%) was more prevalent when compared to subtype B counterpart. A collection of primary mutation in a range of 3 to 1% could be observed in RT most of all related to NNRTI resistance (L100V, K101E, K103N/E, V179A, V179D and Y181C). We could also observe some polymorphic substitutions in drug resistance codons not associated to resistance. A lysine to asparagine substitution was observed at position 65 (K65N) of the RT with a high prevalence (10.4%). However this polymorphism was not associated to tenofovir (TDF) resistance. Other polymorphic sites in RT position 67, 69 and 151 could be observed although these are not related to resistance associated mutations (RAMs; Fig 1).

### Analysis of patients exposed to antiretroviral therapy

**Fig 2:**
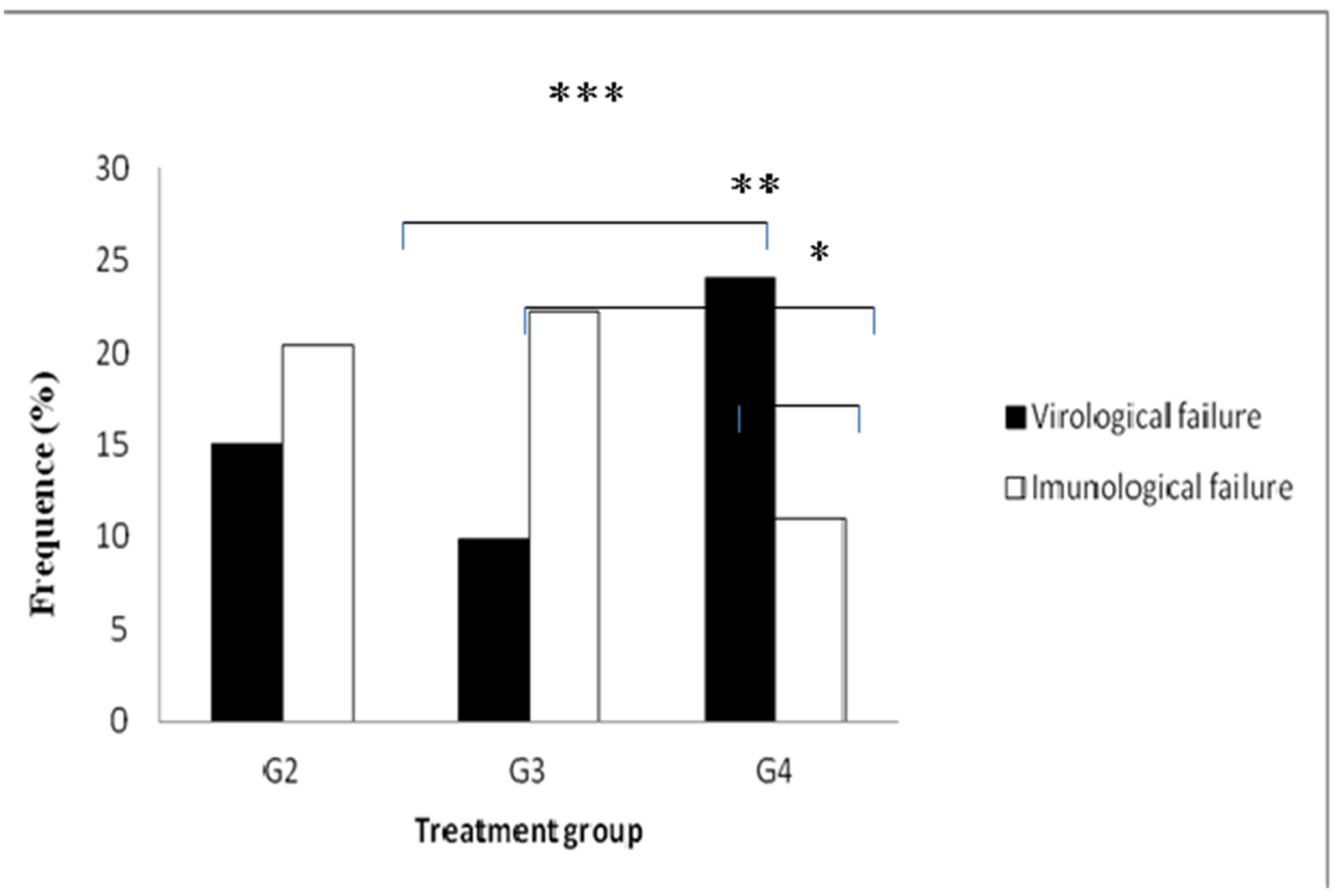
Prevalence of virological and immunological failure in HAART exposed groups (2 to 4). The lines above bars represent significant differences (p<0.05) calculated by *Mann-Whitney* test. ***Significant differences in IF between G3 and G4; **Significant differences in VF between G3 and G4; * Significant differences between IF and VF in G4.

The first-line option widely used in country was based on 3TC+d4T+NVP (87.1%) followed by 3TC+d4T+EFV (9.7%) and 3TC+AZT+EFV (3.2%). (S1 Fig). Maximum virological suppression (<50 copies/ml) was observed in G3 (90.1%) and significant intragroup differences between VF and IF were observed in G4 (p = 0.026). Similarly, significant differences were seen in VF and IF between G3 and G4 (p = 0.023; p = 0.013, respectively;. Fig 2).

### Drug resistance mutations in treated population

A total of 36 (78%) sequences were successfully genotyped from a total of 46 with detectable viral load in ARV treated groups. Resistance profile of sequences from 17 subjects harboring at least ≥1 RAM are documented in Table 2. The average number of RAMs per sequence was 2.7 (range: 1–6). Using immunological criteria to define therapeutic failure only 4 (25%) subjects with RAM were identified. Conversely, virological criteria based on WHO cut-off for determining VF identified 8 (50%) subjects with RAM. In fact, all subjects were with RAM were identified if cut-off was lowered to 50 copies/ml. On the other hand, WHO virological cut off (VL> 1000 copies/ml) was able to detect 80% of subjects with more complex RAM profiles including at least one thymidine analogue mutations (TAMs). However, lower viral loads cut off were associated with less complex patterns of RAM.

**Table 2:** Imuno/virological and drug resistance profile of subjects from differentgroups failing first-line HAART.

Sequence	Time on	Viral Load	T CD4+ count	RAM's	
Code	HAART	(Copies/ml)	(Cells/mm^3^)	ITRN	ITRNN
**B065**	G2 (06–12 mo)	10693	86	M184V	Y181C
**B068**		574123	489		K103N
**C031**	G3 (12–24 mo)	*2833	299		E138A
**C042**		*3209	491	M184V	E138A, G190A
**C056**					G190A
**C060**		*116	645		G190A
**C098**		*4169	527	M184V, K219E	K103N, Y181C
**D001**	G4 (24 < mo)	*364	253	M184V	V108I, Y181C
**D016**		252408	142	M184V, T215F	K103S, G190A
**D030**		57135	130	D67N, M184V	K101E, Y181C
**D032**		13910	267	D67N, T69A	A98G, K101Q, V106A
**D033**		*586	252	M184V	K103N, G190A
**D043**		*2616	404	M184V	G190A
**D049**		100632	548	M184V	
**D058**		33949	210		G190A
**D078**		157784	103	M41L, M184V, L210W, T215Y	V108I, Y181C
**B105**		*138	329	M184V, T215N, K219R	K103S, G190A

* Sample with load viral below of 5000 copies/ml, and with resistance mutations.

Analysis of RAMs showed that 70.6% of the subjects harbored RAM´s associated to NRTIs and 84.1.% to NNRTIs. The most frequent NRTI and NNRTI mutations found were M184V (91.7%) and G190A (50%), respectively. TAMs were mainly seen in sequences from G4 (n = 5; 50%), with one occurrence in G3 (25%). These TAMs were mainly belonged to TAM-2 pathway (83.3%). The most common NNRTI mutations observed were K103N/S and G190A. More complex RAM patterns were seen with increasing exposure to HAART. For example, in G4 there were sequences incorporating >3 NRTI mutations (20%), and in G3 and G4 there were sequences carrying >2 NNRTI mutations (50%) (Table 2). Since all patients were in first line therapy, no major PI mutations were observed, although secondary mutations already described to be subtype C polymorphic sites were identified probably related to subtype C molecular signatures (S2 Fig).

### Susceptibility to RT inhibitors based on genotypic data

**Fig 3:**
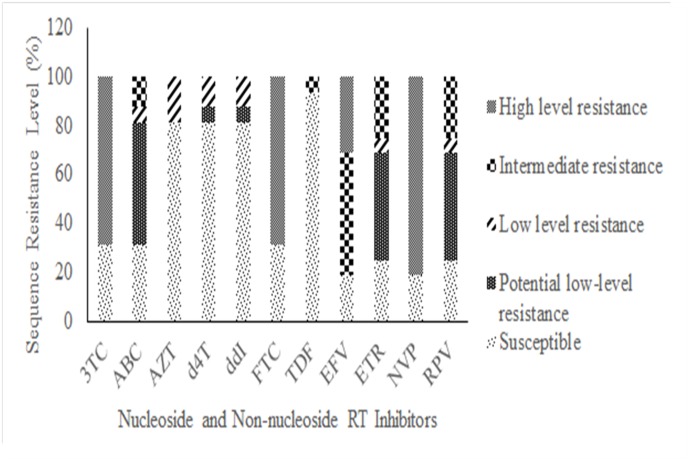
Prediction of drug viral susceptibility to nucleoside reverse transcriptase inhibitors (NRTI) and nonnucleoside reverse transcriptase inhibitors (NNRTI), of sequences harboring at least one RAM from all groups (n = 17). Drug susceptibility predicted using Stanford HIVdb (Version 6.2.0).

Analysis of 17 sequences with RAMs profile showed a decrease in drug susceptibility to all approved RT inhibitors with an accentuated decrease to NRTI inhibitors 3TC and emtricitabine (FTC). Similarly, the genotypic resistance to abacavir (ABC) was observed in 70% of sequences analyzed however, resistance level ranged from potential low to intermediate level of resistance. Prediction of susceptibility to the remaining NRTI inhibitors showed that solely 20% of isolates decreased their response to AZT, d4T and DDI whereas the majority of sequences in this group remain susceptible to TDF (90%). Additionaly, the majority of samples analyzed shown a high level resistance to EFV and NVP and these isolates carry a complex pattern of NNRTI-RAM such as G190A, K103N, and Y181C. Due to this complex pattern, susceptibility to second generation NNRTIs, rilpivirine (RPV) and etravirine (ETR) showed some level of cross-resistance that ranged from potential low level to intermediate resistance based on Stanford HIVDB prediction algorithm (Table 2 and Fig 3).

## Discussion

The present study shows the prevalence of RAM’s and the mutation profile in drug naive individuals in Mozambique and subjects failing HAART attending public health care services.

Current treatment strategies have shown to be effective in treating HIV infection in Mozambique where the epidemic is dominated by subtype C, as reported previously [21]. In fact we observed that 84.1% of individuals in all treatment groups had undetectable level of viremia in plasma when sampled. The HAART success rate after 12 months was 84.9%, and this is comparable to previously documented studies on non-B subtypes after 12 months on HAART, and this value is in agreement with WHO target for HIVDR prevention, defined by VL suppression ≥70% [25,26].

Our results suggest that maximum viral suppression is observed after 12 months even if we consider that our patients has initiated HAART severely immunocompromised. It was demonstrated that current drug regimens suggested by WHO for first-line treatment approach are effective in promoting immunological recovery as given by significant increase in median TCD4+ count from G2 (291cells/mm^3^) to G4 (383 cells/mm^3^), an 92 cells/mm^3^ increase between these two groups. Nonetheless, this increase is bellow the values reported by Auld and colleagues on data from Mozambique where the mean gain was 186, 273 and 293 cell/mm^3^ for a HAART period of 24, 36 and 48 months respectively [27]. Statistically non significant increase was observed between G2 and G3 that may be justified by biphasic characteristic of the immune reconstitution period, and by the fact that the majority of Mozambican subjects initiate HAART with low TCD4+ counts (bellow 200 cells/mm^3^). The fact that this study used a cross-sectional approach may also justify such low increases observed [28–30].

RAM’s were identified in subjects in group of patients with viremia levels below the cut-off value preconized by WHO to consider a virological failure (3500 copies/ml). Of note, there is the low concordance between viral detectable VL and RAM in G2 that may result from high viral loads at the beginning of treatment and after 12 months on HAART the viral replication is still been suppressed. Drug resistance patterns were in accordance with the treatment options used in the country. High prevalence of mutation M184V reflects the wide use of 3TC. The occurrence of this mutation in early treatment groups may explain low prevalence of TAMs in these groups [31]. TAMs were shown to occur mainly in subjects failing treatment with very low TCD4+ counts, thus rendering the immunological criteria for identifying treatment failure effective in this context. However, our results show that subjects failing therapy with less complex patterns of RAM´s tend to have relatively high TCD4+ counts. All together these results confirm previous reports where more complex patterns of RAM´s are observed with increasing exposure to HAART and these are not easily detected using immunological criteria solely [32,33]. Mutations K103N and G190A were common in all treatment groups, reflecting the wide initial use of EFV followed by NVP, as tuberculosis have been shown to be frequent in subjects initiating HAART (11%) [26]. Interestingly, we observed that despite the fact that 87% of subjects were receiving d4T, and the risk of emergence of K65R mutation associated to subtype C [18], mutation K65R was not common in subjects on a mean treatment time of up to 45 months. Surprisingly, a prevalence of 10.4% of the mutation K65N was observed in naïve subjects. This mutation does not seem to occur in people on HAART treatment [34,35], and to a lesser extent in drug naïve individuals [36] Nonetheless, it was seen that K65N has similar drug resistance profile as a virus with arginine (Arg) substitution [37]. Interestingly, Chunduri and colleagues have seen that a K65N virus has a less replicative capacity and processivity, when compared to a K65R [38]. All together these may explain the absence of mutation K65N in subjects failing HAART in our dataset, as these are poorly selected in during treatment. As current WHO guidelines recommend the substitution of d4T by ZDV or TDF, [2] this observation along with the observation that between 80–90% of virus sequences from subjects with at least one RAM high are still susceptible to these drugs along with DDI. Additionally, with the introduction of new generation NNRTIs, debates are carried in order to decide if these drugs should be used in resource-limited countries, where subjects are heavily exposed to NVP and EFV. Our results show that only 20% of the sequences exhibiting RAM´s to EFV and NVP remain susceptible to these drugs. Then, caution should be taken if these second generation NNRTIs are introduced as subjects carrying RAMs may be already fully resistant to RPV and ETR, and the absence of viral load monitoring may make the identification of not suppressive therapies hard to identify.

Low prevalence of primary drug resistance based on WHO mutation list was observed in our data set and this was consistent with results recently published by our group for the same geographic area, using the WHO threshold survey methodology. The dynamics of the epidemic differ across the southern, central and northern region of Mozambique. Recent data from our group have shown that primary RAM tend to show the same trend, with the central region exhibiting the highest prevalences (unpublished data). All together with the expansion of HAART program in the country indicate that efforts should be put in place to monitor the emergence of RAM in naïve subjects, as this may ensure the use of fully suppressive drugs in the country. Furthermore, constant evaluation of treatment program and gradual introduction of viral load to monitor treatment, at least in subjects exposed to HAART for longer, will ensure timely detection of treatment failures, preventing the occurrence of TDR and new infections. In vitro selection culture should be performed in order to generate data describing the outcome of viruses carrying mutation K65N with and without TDF.

Of note, mutation T74S has been described as polymorphic in subtype C viruses circulating in Africa and South America, and it is associated with reduced nelfinavir susceptibility [39], here we showed the presence of this mutation at high prevalences in HAART naïve populations. Similarly, further studies should be performed in order to evaluate the impact of such polymorphisms on second-line based HAART.

## Supporting Information

S1 Fig Distribution of different first-line treatment options in treated patients. (TIF)

S2 Fig Prevalence of HIV-1 DRM and aminoacid substitutions at codon positions known to be associated to resistance to protease inhibitors in 17 sequences from naïve subjects, according to IAS-USA 2011 list. (TIF)
