# Supplementary figures and images for: Trends in Prevalence of HIV-1 Drug Resistance in a Public Clinic in Maputo, Mozambique
Source: PLoS One. 2015 Jul 7;10(7):e0130580. doi: 10.1371/journal.pone.0130580 (PMC4494809; doi:10.1371/journal.pone.0130580)

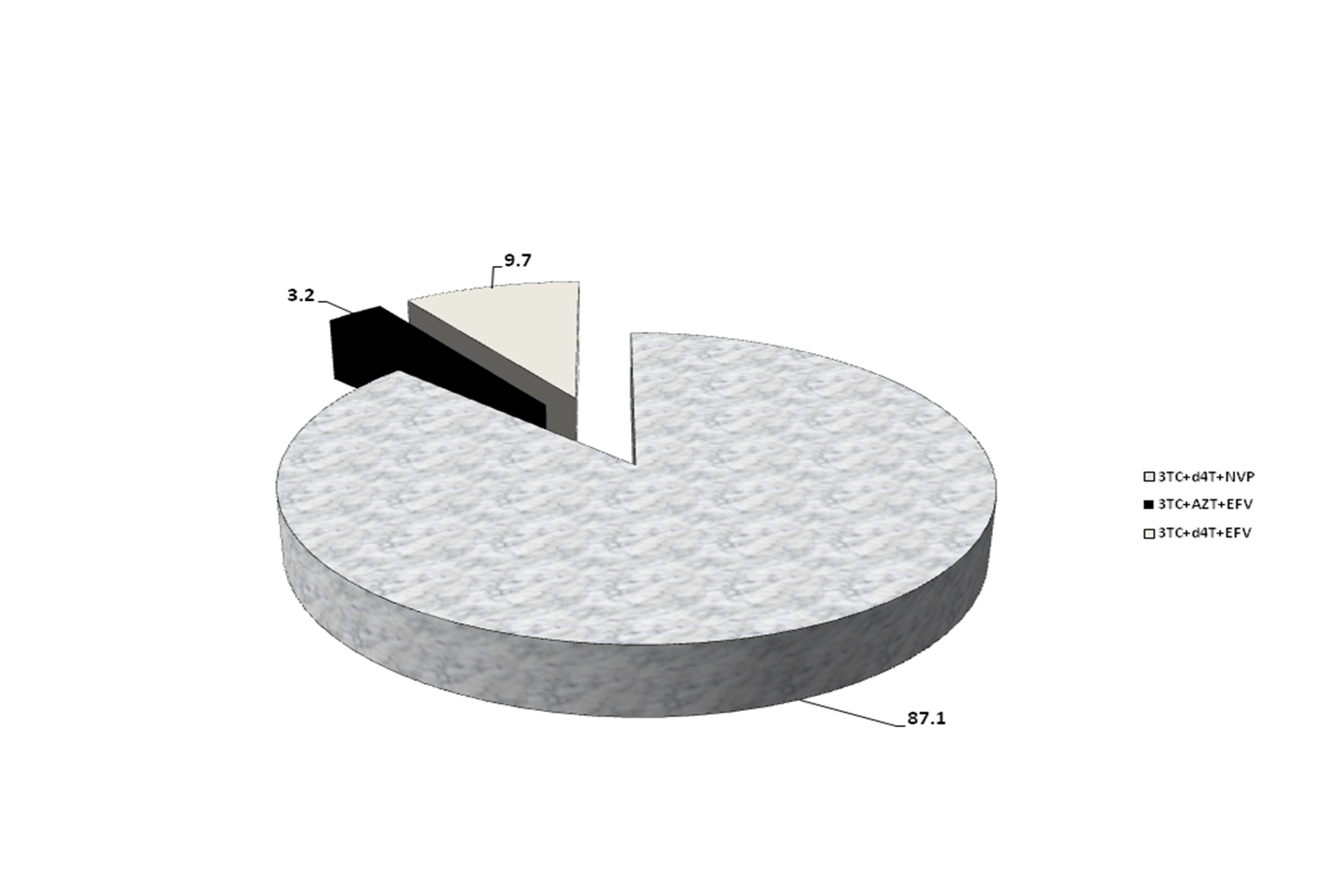

Supplement: S1 Fig — (TIF) [file pone.0130580.s001.tif]

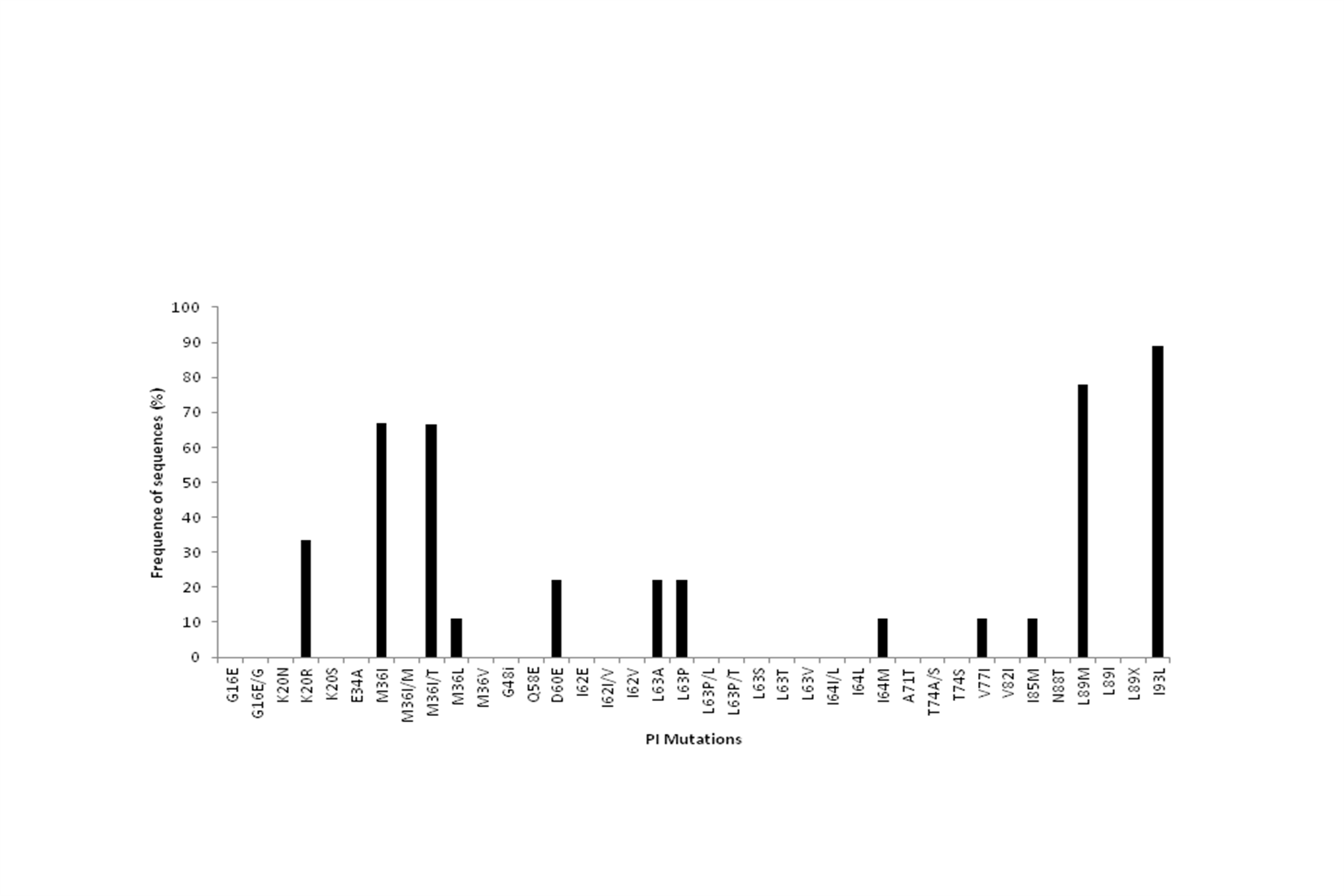

Supplement: S2 Fig — (TIF) [file pone.0130580.s002.tif]
